# Untying the Next Genetic Thread in a Family With MEN2A Syndrome: A Case Report

**DOI:** 10.1002/ccr3.71065

**Published:** 2025-10-10

**Authors:** Riyaz Shrestha, Mohammad Adnan Adil, Binita Basnet, Himal Karki, Pukar Maskey, Samir Shrestha, Mohammad Nasim Alam

**Affiliations:** ^1^ Department of Surgery Patan Academy of Health Sciences Lalitpur Nepal

**Keywords:** genetic screening, medullary thyroid carcinoma, MEN2A, partial adrenalectomy, pheochromocytoma

## Abstract

Multiple endocrine neoplasia type 2A (MEN2A) is a rare autosomal dominant syndrome characterized by medullary thyroid carcinoma (MTC), pheochromocytoma, and primary hyperparathyroidism. Early genetic screening is crucial for timely intervention. We report a familial case of MEN2A involving four affected members across two generations. The index patient, a 40‐year‐old male with prior MTC and pheochromocytoma, presented with recurrent adrenal disease. His 41‐year‐old sister was diagnosed with MTC and pheochromocytoma despite negative genetic testing. The youngest sibling (37 years) and the index patient's 18‐year‐old son were diagnosed with MTC, with the former testing positive for a RET mutation. All underwent appropriate surgeries with ongoing surveillance. This case highlights the variable clinical presentation and genetic penetrance of MEN2A within a single family. It underscores the importance of genetic screening in all MTC patients and their first‐degree relatives, as early identification of asymptomatic carriers enables timely prophylactic interventions.

**Summary**

- This case report describes a family with medullary thyroid carcinoma (MTC) across two generations, alongside varying MEN2A syndrome features.
- It highlights the importance of family‐wide genetic screening in MTC cases and the role of early diagnosis and treatment (organ‐preserving surgeries like partial adrenalectomy) in reducing morbidity, mortality, and improving long‐term outcomes.

## 1 Introduction

Multiple endocrine neoplasia type 2A (MEN2A) is a rare syndrome with an autosomal dominant mode of inheritance and is characterized by tumors of the thyroid and adrenal glands, and primary hyperparathyroidism [1]. The prevalence of MEN2A syndrome is 1 in 25,000 people and up to 95% have a germline mutation in the rearranged during transfection (RET) protein gene at chromosome 10 [1, 2]. Medullary thyroid carcinoma (MTC) is the most common manifestation, occurring in up to 100% of the patients with MEN2A syndrome. Pheochromocytoma occurs in about half of the patients, and up to 25% of patients suffer from primary hyperparathyroidism [1]. A delay in the diagnosis and treatment of MTC results in the development of metastatic disease and significantly increases morbidity and mortality in MEN2A syndrome [3].

In MEN2A syndrome, autosomal dominant mode of inheritance allows parents to transmit the gene to their offspring with 50% probability. But, due to variable penetrance of the syndrome, clinical presentation varies among affected family members [2]. The variability in the presentation, morbidity associated with MTC, and its commonality in MEN2A syndrome highlights the need for family‐wide genetic screening in case of MTC [3]. In addition to that, early diagnosis of asymptomatic carriers helps in risk stratification, timely prophylactic surgeries, and surveillance of the disease progression to reduce morbidity and mortality associated with it [4].

## 2 Case History/Examination

A 40‐year‐old male, index case with a history of subtotal thyroidectomy performed 12 years ago for medullary thyroid carcinoma (MTC) and open right adrenalectomy with excision of a preaortic paraganglioma 5 years ago for histologically proven pheochromocytoma, had revisited as a case of uncontrolled hypertension despite being on four antihypertensive medications. However, he was lost to follow‐up for 2 years and had returned to the outpatient department (OPD) after his elder sister developed similar symptoms. On examination, he had no palpable thyroid mass, and his thyroidectomy scar was well appreciable.

His 41‐year‐old sister, the eldest of three siblings, presented to the OPD with a painless neck swelling that had persisted for 20 years. On examination, she had bilateral thyroid nodules with no signs of local invasion or compression.

The youngest sibling of the index patient, a 37‐year‐old male, presented with a painless, progressively enlarging left‐sided neck swelling for 7 months. On examination, it was found to be hard, non‐tender, and without signs of local invasion or compression.

His 18‐year‐old son had also presented with a painless, gradually progressive left‐sided neck swelling for 3 months. On examination, his son had a firm, 2 cm × 2 cm swelling in the left thyroid lobe, with no overlying skin changes.

## 3 Management

**FIGURE 1:**
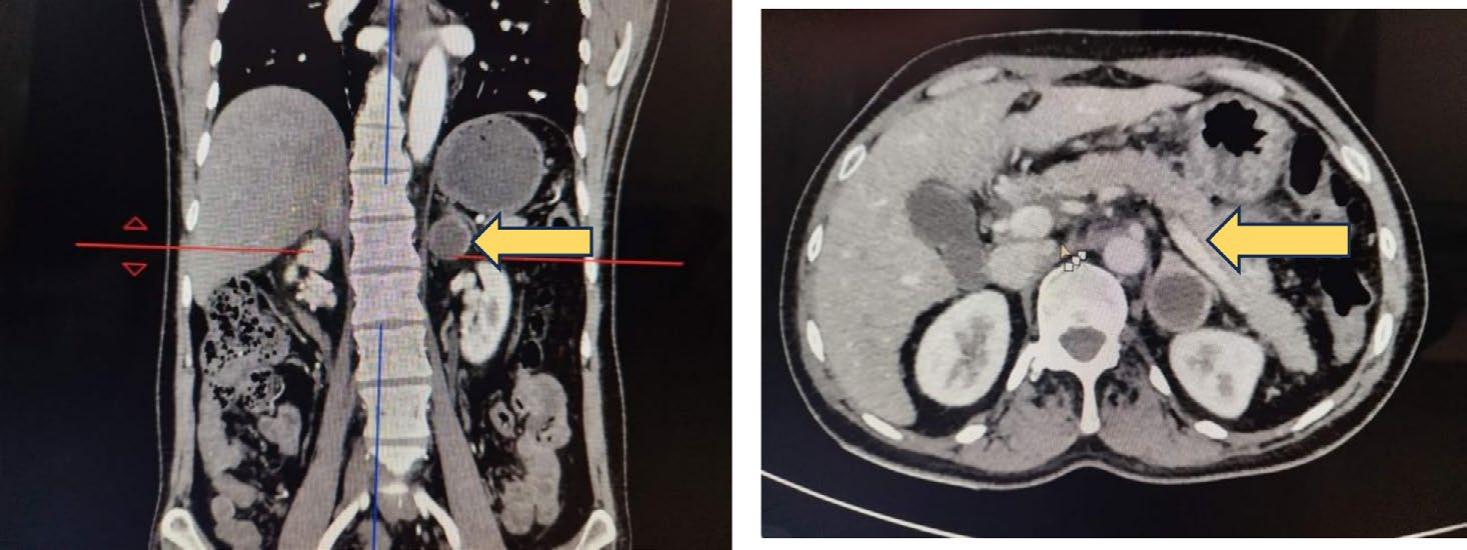
Follow‐up contrast enhanced computed tomography (CECT) of abdomen and pelvis of index case showed left adrenal lesion measuring 28 mm × 27 mm × 26.3 mm (yellow arrowhead).

**FIGURE 2:**
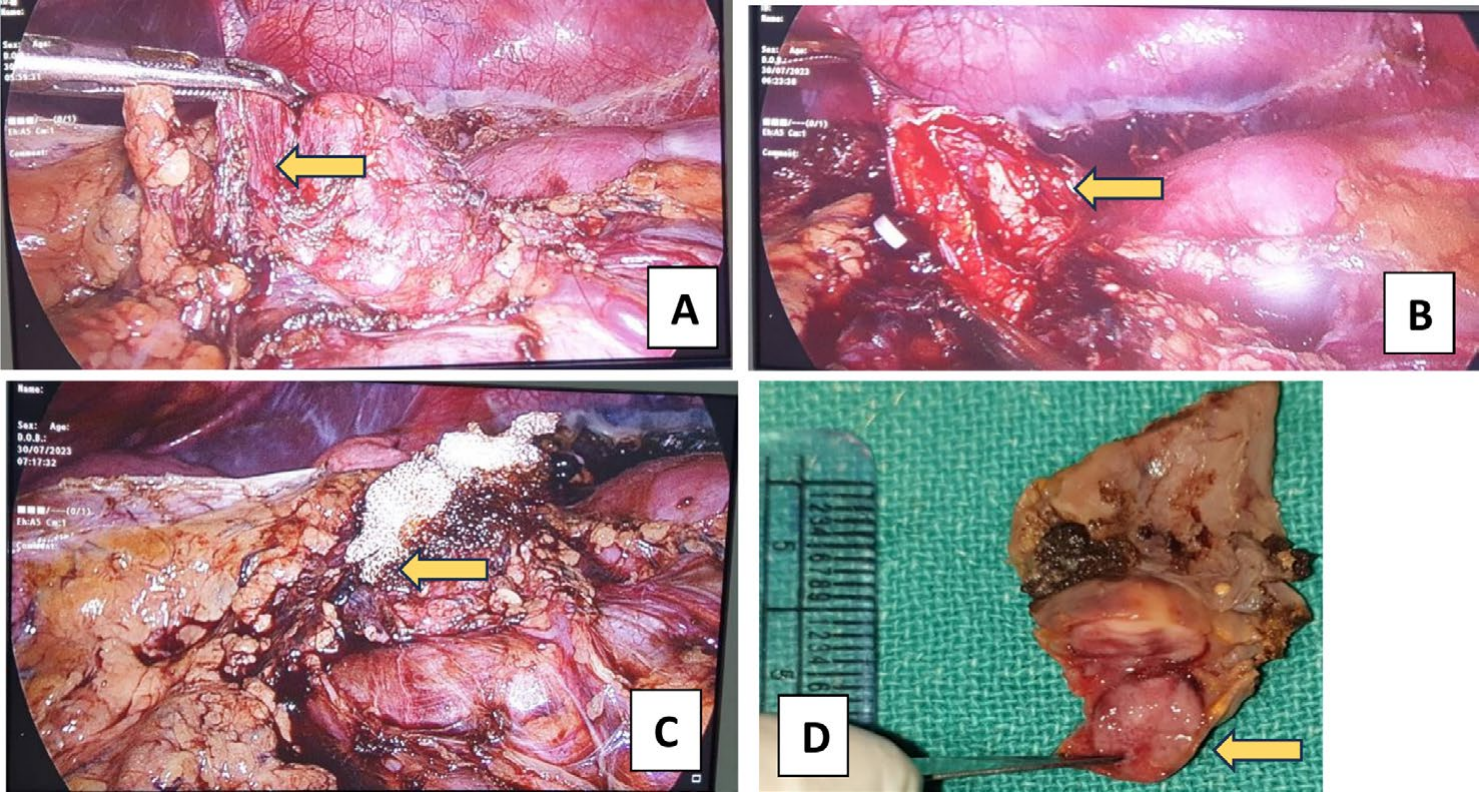
Intraoperative (A–C) and postoperative (D) pictures showing laparoscopic resection of the left adrenal gland and the resected adrenal gland, respectively. Note that the adrenal gland was not completely resected.

The abdominal ultrasonography (USG) of the index patient revealed a cystic space‐occupying lesion in the left adrenal gland measuring 2.5 cm × 2.7 cm. Corroborating with this, the contrast enhanced computed tomography (CECT) of the abdomen and pelvis revealed a new left adrenal lesion measuring 2.8 cm × 2.7 cm × 2.63 cm, consistent with a pheochromocytoma (Figure 1). Biochemical testing showed elevated 24‐h urinary metanephrine of 1520.96 mcg/24 h (normal: 25–312 mcg/24 h), normetanephrine of 956.17 mcg/24 h (normal: 35–445 mcg/24 h), and serum calcitonin level of 245 pg/mL (normal: < 8 pg/mL), raising suspicion of recurrence. Therefore, he underwent laparoscopic left partial adrenalectomy to remove the newly detected adrenal lesion while preserving adrenal function (Figure 2).

The eldest sibling of the index patient that is, the 41 years old lady's neck USG revealed a bilateral nodular goiter. Fine needle aspiration cytology (FNAC) showed Bethesda category IV (suspicious for follicular neoplasm). Plasma‐free metanephrine and normetanephrine levels were 1160 pg/mL (normal: 7.9–88.7 pg/mL) and 3460 pg/mL (normal: 20.1–135.4 pg/mL) respectively, suggesting a possible pheochromocytoma. CECT of the abdomen and pelvis revealed a heterogeneously enhancing solid‐cystic lesion in the right adrenal gland, measuring 4.7 cm × 4.3 cm × 4.1 cm. However, her genetic testing for MEN2A syndrome came out to be negative. She underwent laparoscopic right adrenalectomy, with the excision of a 5 cm adrenal tumor, preserving a small rim of normal adrenal cortex. Following adrenalectomy, she also underwent total thyroidectomy with bilateral selective lymph node dissection (Levels II, III, IV, and V) and central compartment clearance to treat her thyroid neoplasm.

Neck USG of the index patient's youngest sibling showed a large left thyroid mass. FNAC confirmed MTC, and biochemical markers revealed serum calcitonin of 652 pg/mL (normal: < 18.2 pg/mL) and carcinoembryonic antigen (CEA) of 286 ng/mL (normal: < 3 ng/mL), while plasma‐free metanephrine was < 7 pg/mL (normal: 7.9–88.7 pg/mL), ruling out adrenal involvement at presentation. Genetic testing identified a pathogenic variant in exon 11 of chromosome 10 in the RET gene in a heterozygous state, confirming hereditary MEN2A syndrome. Therefore, he underwent total thyroidectomy with bilateral selective node dissection and central compartment clearance.

The index patient's son's neck USG revealed multiple nodular lesions in the left thyroid lobe, the largest measuring 1.3 cm × 1.9 cm. FNAC confirmed MTC (Bethesda Category VI). CEA and 24‐h urinary metanephrines were 87.3 ng/mL (normal: < 3 ng/mL) and 376.47 mcg/24 h (normal: 25–312 mcg/24 h), respectively, while thyroid function test (TFT) and calcium levels were within normal limits. CECT of the abdomen and pelvis identified a hypodense nodule in the lateral limb of the left adrenal gland, measuring 9 mm × 7 mm, suggestive of a lipid‐poor adrenal adenoma. Therefore, he underwent laparoscopic left partial adrenalectomy, where a 1 cm × 1 cm adrenal mass was excised.

None of the family members had features suggestive of hyperparathyroidism.

## 4 Conclusions and Results

Postoperatively, all patients were placed under regular biochemical and radiological surveillance. The index patient's left adrenal lesion showed stability post‐surgery, with no evidence of recurrence. The 41‐year‐old sister recovered well, with regular monitoring for residual or recurrent disease. The 37‐year‐old youngest sibling remained stable, with continued follow‐up for potential adrenal involvement. The 18‐year‐old son was scheduled for long‐term surveillance, given his early presentation with MTC and adrenal involvement.

## 5 Discussion

MEN syndromes are rare syndromes with autosomal dominant inheritance, meaning a child has a 50% chance of inheriting the condition from an affected parent. There is a variable lifetime penetrance for the manifestation of medullary thyroid carcinoma, pheochromocytoma, and primary hyperparathyroidism. It occurs due to a germline mutation in the RET proto‐oncogene, located on chromosome 10 (10q11.2), composed of 20 exons that encode a tyrosine kinase transducing signals for cell growth and differentiation [1]. The majority of MEN2A syndrome has germline RET mutations in exon 10 or exon 11, although rare mutations in exons 13, 14, and 15 can also be causative [5]. So, negative genetic testing that usually tests for the common exon mutations does not rule out the presence of a rare RET mutation outside of the sequenced regions, like in our index case's elder sister.

**FIGURE 3:**
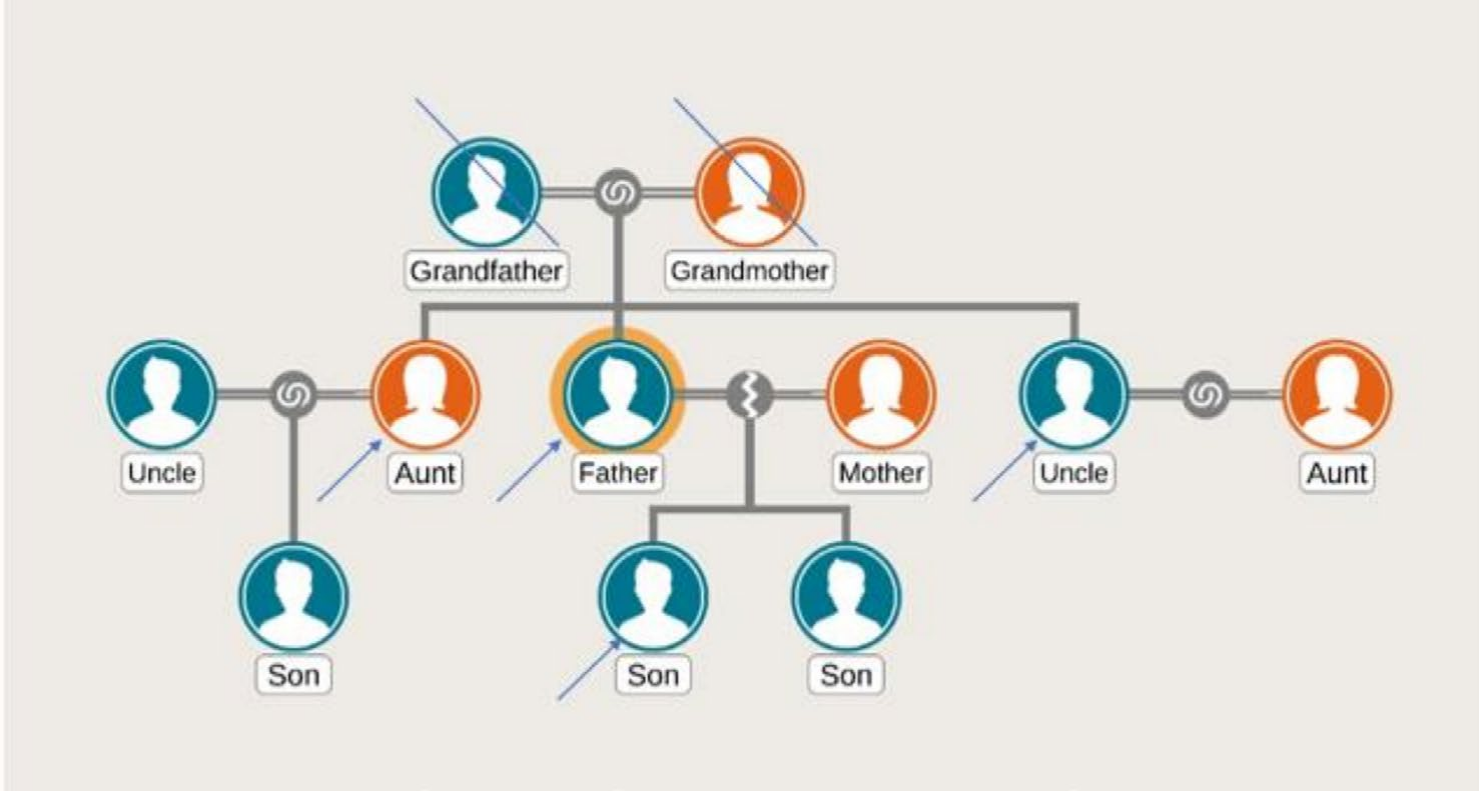
Genealogy showing 4 family members across 2 generations affected by multiple endocrine neoplasia Type 2A (MEN2A) syndrome.

Much like the family afflicted by MEN2A syndrome in our case (Figure 3), a 3‐generation family in India was found to have its 13 members out of 21 manifesting the traits, with almost all of them (12 out of 13) found to have a RET mutation. All the 13 family members had MTC, 10 family members had pheochromocytoma and 4 developed lymph nodal recurrence during follow‐up, for which they underwent reoperations, with a median duration of recurrence being 48 months [6]. MTC happens to be the most common manifestation in MEN2A syndrome and also the most common cause of mortality and morbidity in the afflicted patients. Owing to this fact, alongside 25% of the MTC running in families, it's recommended that all patients with MTC be screened for pathogenic variants in the RET proto‐oncogene [3].

The rule of 25% cuts both ways, as 25% of pheochromocytomas are found to be familial; especially those with a positive family history, those presenting earlier than 50 years of age, and those with multiple, malignant, or bilateral lesions. So, it's recommended that only these patients with pheochromocytoma, along with their first‐degree family members, be screened for familial syndrome, including MEN2A syndrome [7]. Also, partial adrenalectomy could be a suitable option for patients with hereditary conditions like MEN2A syndrome, as there is a higher chance of disease developing in the contralateral adrenal gland in the future [8].

A study published by Machens et al. [9] in 2020 revealed that MEN2A associated neoplasms were diagnosed significantly earlier over time, as evidenced by a decline in the median age at surgery. For instance, in the first adrenal gland, from 51 years in birth cohort 1922–1950 to 22.5 years in birth cohort 1991–2000; and in the second adrenal gland, from 51 years in birth cohort 1922–1950 to 29.5 years in birth cohort 1981–1990. This study highlights the significance of early and regular biochemical and genetic screening in detecting adrenal tumors earlier with a resultant decline in the diameter of the adrenal tumor at presentation, thereby leading to the widespread adoption of partial adrenalectomies.

When it comes to the role of prophylactic thyroidectomy in MEN2A syndrome, the American Thyroid Association (ATA) recommends carrying out thyroidectomy in MEN2A patients aged 5 years and below with the most common mutation (codon 634); the benefits of which overpower the lower incidences of risks of hypoparathyroidism and impaired brain development and retarded growth [10].

## Author Contributions

**Riyaz Shrestha:** conceptualization, formal analysis, investigation, methodology, project administration, resources, visualization, writing – original draft, writing – review and editing. **Mohammad Adnan Adil:** conceptualization, data curation, investigation, supervision, validation, visualization, writing – original draft, writing – review and editing. **Binita Basnet:** formal analysis, investigation, methodology, writing – original draft, writing – review and editing. **Himal Karki:** formal analysis, investigation, methodology, supervision, writing – original draft, writing – review and editing. **Pukar Maskey:** conceptualization, investigation, methodology, supervision. **Samir Shrestha:** conceptualization, investigation, methodology, project administration, supervision. **Mohammad Nasim Alam:** conceptualization, investigation, methodology, supervision.

## Ethics Statement

The authors have nothing to report.

## Consent

Written informed consent was obtained from the patient to publish this report in accordance with the journal's patient consent policy.

## Conflicts of Interest

The authors declare no conflicts of interest.

## Data Availability

The data that support the findings of this study are available from the corresponding author upon reasonable request.
